# Novel Diagnostic Biomarkers Related to Oxidative Stress and Macrophage Ferroptosis in Atherosclerosis

**DOI:** 10.1155/2022/8917947

**Published:** 2022-08-05

**Authors:** Minhui Li, Siyuan Xin, Ruiyuan Gu, Lin Zheng, Jie Hu, Ruijing Zhang, Honglin Dong

**Affiliations:** ^1^Second Hospital of Shanxi Medical University, Taiyuan, Shanxi, China; ^2^Baotou Medical College, Inner Mongolia, China

## Abstract

Atherosclerosis (AS) is a chronic inflammatory disease, which has a complex interplay between altered immune metabolism and oxidative stress. Therefore, we aimed to determine the oxidative stress and immune-related biomarkers in AS. Differential gene expression analyses are based on the GSE100927 dataset in the Gene Expression Omnibus (GEO), and 389 oxidative stress (OS) genes are identified based on gene set enrichment analysis (GSEA). We identified 74 differentially expressed genes related to oxidative stress (DEOSGs). “CIBERSORT” and “WGCNA” R Packages were used to compare the differences in immune infiltration levels between AS and control samples. The DEOSGs (*N* = 74) were intersected with the key module's genes of WGCNA (*N* = 972), and 27 differentially expressed immune-related oxidative stress genes (DEIOSGs) were obtained. To identify the pivotal genes, a protein-protein interaction (PPI) network was constructed using the STRING database and the Cytoscape software. *MMP9*, *ALOX5*, *NCF2*, *NCF*, and *NCF4* were identified as diagnostic markers of AS, and we validated them in the GSE57691 dataset. The expression levels of the five diagnostic genes were significantly highly expressed in the AS group. Correlation analysis and single-cell analysis revealed that five diagnostic genes were mainly correlated with macrophages M1. We, respectively, intersected differentially expressed genes (DEGs) with ferroptosis gene set, necroptosis gene set, and pyroptosis gene set. The findings suggested that *ALOX5* and *NCF2* were differentially expressed genes of ferroptosis. High expression of five hub genes in RAW264.7 macrophages were confirmed by PCR. High *ALOX5* and *NCF2* expression levels in plaque tissues were confirmed by immunohistochemistry (IHC) and western blotting. Our study identified that *MMP9*, *ALOX5*, *NCF2*, *NCF1*, and *NCF4* were diagnostic genes of AS and associated with oxidative stress. *ALOX5* and *NCF2* may be involved in the formation of the necrotic core in AS by regulating macrophage ferroptosis.

## 1. Introduction

Multiple cardiovascular and cerebrovascular diseases, including coronary heart disease, ischemic stroke, and peripheral artery disease, dominate global mortality and disability statistics [1]. Atherosclerosis (AS) is a crucial pathological mechanism of these diseases, characterized by the accumulation of low-density lipoprotein (LDL) particles in large- and medium-sized arteries, migration of mononuclear cells and other immune cells through dysfunctional endothelial cells, and lipid plaque formation. With the release of inflammatory factors, a chronic inflammatory response in the arterial wall occurs [2, 3]. Plaque rupture, thrombosis, and lumen narrowing obstruct blood flow, leading to a series of major adverse cardiovascular events (MACEs). For the treatment of atherosclerosis, the most used drugs are currently statins, which lower LDL cholesterol levels. These therapies, however, have not been effective in reducing levels of MACESs [4]. Therefore, understanding the etiology and pathogenesis of AS can guide the clinical diagnosis and therapies and improve clinical outcomes.

In recent years, many researchers have attempted to combine immunological and anti-inflammatory treatments and reduce MACEs. For instance, canakinumab, which targets to the interleukin-1*β* (IL-1*β*) innate immunity pathway, can significantly reduce MACEs [5]. Moreover, research has shown that immune checkpoint proteins and costimulatory molecules play a substantial role in regulating atherosclerosis [6, 7]. These studies provide novel insights into the significance of immune modulation for AS. In addition, plaque formation results from interactions between immune cells and oxidative stress (OS). In the arterial wall, increased oxidative stress can promote the accumulation of modified lipoproteins, alter macrophage metabolism, and lead to proatherosclerotic immune cell infiltration [8]. An oxidative stress state is marked by elevated levels of reactive oxygen species (ROS). Cardiovascular risk factors, such as hypertension, hypercholesterolemia, and hyperlipidemia, can promote ROS production. But few studies have been conducted to explore the combination of oxidative stress and immune infiltration in AS.

Immune infiltration and oxidative stress play important roles in AS. In this study, we conducted a systematic bioinformatics analysis to outline the immune infiltration landscape and combined oxidative stress to determine the diagnostic genes of AS. Additionally, we deliberated the relationship between ferroptosis and infiltrating immune cells to gain a better understanding of the potential molecular process during the development of AS.

## 2. Materials and Methods

### 2.1. Data Source

In this study, we obtained a gene expression microarray from the Gene Expression Omnibus (GEO) database (http://www.ncbi.nlm.nih.gov/geo). The microarray data were obtained from the GPL17077 with the accession number GSE100927 (AS = 69, control = 35), GPL10558 platform with the accession number GSE57691 (AS = 9, control = 10), and GPL18573 platform with the accession number GSE159677. GSE100927 and GSE57691 were used as training set and external validation sets, respectively. GSE159677 was used as a single-cell set. We obtained 389 OS-related genes from the gene set enrichment analysis (GSEA) [9].

### 2.2. Identification of DEGs

The differentially expressed genes (DEGs) from GSE100927 were identified using the Limma R package on normalized count data. The parameters |Log2fold change| >0.5 and adj. *P* < 0.05 were used as the screening criteria for DEGs. Moreover, heatmap and volcano plot of DEGs from the databases were constructed using pheatmap and ggplot2 R packages.

### 2.3. KEGG and GO Enrichment Analyses

To reveal the potential biological functions and underlying mechanisms of genes, we used the R package “clusterProfiler” to analyze Gene Ontology (GO) and Kyoto Encyclopedia of Genes and Genes (KEGG) term enrichment of the target genes [10]. GO terms, including biological processes (BPs), cellular components (CCs), molecular functions (MFs), and KEGG pathways with adjusted *P* < 0.05, were considered statistically significant.

### 2.4. Immune Infiltration Analysis

The proportions of the 22 types of immune cells in samples from GSE100927 were obtained using the CIBERSORT algorithm [11]. The “vioplot” package was used to compare the levels of 22 types of immune cells between AS and control samples.

### 2.5. Construction of Weighted Gene Coexpression Networks

In this study, the R package “WGCNA” [12] was used to construct the weighted gene coexpression network analysis (WGCNA). First, hierarchical clustering was performed on the study samples to detect the outliers and remove the abnormal samples. Second, to build a scale-free network, soft powers of *β* = 2 were selected using the function pickSoft Threshold. Thereafter, the adjacency matrix was established and converted to a topological overlap matrix (TOM), and the gene dendrogram and module color were established using the degree of dissimilarity. The correlations between modules and differentially infiltrating immune cells were then calculated using the WGCNA package. Modules with high correlation coefficients were considered as candidates related to differentially infiltrating immune cells and selected for subsequent analyses. With the candidate module selected, we defined |MM| (|Module membership|) >0.8 and |GS| (|gene significance|) >0.20 as the screening criteria for filtering key genes in the candidate module. The intersection of differentially expressed genes related to oxidative stress (DEOSGs) and genes in key modules were performed using the “VennDiagram” R package and defined as differentially expressed immune-related oxidative stress genes (DEIOSGs), which were used for subsequent analysis.

### 2.6. Construction of Protein-Protein Interaction Network and Screening of Hub Gene

The protein-protein interaction (PPI) network was constructed using the Search Tools for the Retrieval of Interacting Genes (STRING) database [13]. The Cytoscape software was then used to visualize the PPI network. The molecular complex detection (MCODE) plug-in in the Cytoscape was used to identify significant gene clusters and obtain hub genes.

### 2.7. The ROC Curve Analysis and Expression Analysis

In the GSE100927 dataset, we performed receiver operating characteristic (ROC) curve analysis on each screened hub gene to verify its accuracy. The “pROC” package was used for ROC curve analysis. The hub genes with AUC> 0.7 were deemed useful for disease diagnosis [14]. Expression levels of hub genes between AS and control samples were displayed in the boxplots generated by the “ggplot2” in R package. We analyzed the functional similarity of genes using the “GOSemSim” package in R. The corrplot package was used to analyze the correlation of genes.

### 2.8. Correlation Analysis between Infiltrating Immune Cells and Diagnostic Genes

Immune infiltration analysis was performed using the CIBERSORT algorithm. The corrplot in R was used to calculate the Spearman correlation analysis between infiltrating immune cells and diagnostic genes. We visualized the correlations between diagnostic genes and immune cells with lollipop.

### 2.9. GSEA Analysis

The GSEA was used to identify the potential function of the diagnostic genes. The chosen reference gene set was downloaded from the Molecular Signature Database (MSigDB). A *P* < 0.05 was used as the criterion for significant enrichment.

### 2.10. Single-Cell Analysis

We incorporated atherosclerotic core (AC) plaques and patient-matched proximal adjacent (PA) portion transcriptome data from GSE159677. Subsequently we conducted the single-cell analysis using the “Seurat,” “tidyverse,” and “Matrix” R packages.

### 2.11. Construction of Potential TF- and miRNA-Target Gene Regulatory Networks and Small-Molecule Drug Prediction

The miRNet (https://www.mirnet.ca/) online database was accessed to identify possible miRNA targeting diagnostic genes [15]. The upstream transcription factors (TF) were predicted using the NetworkAnalyst (https://www.networkanalyst.ca/) [16]. Subsequently, the results were visualized using the Cytoscape software. Small-molecule drugs were searched using the gene name on CTD website, after which a serial of drug-gene interaction pairs was obtained. The gene–drug interaction networks were visualized using the Cytoscape.

### 2.12. In Vitro and In Vivo Analyses

RAW264.7 macrophages were cultured. *5-lipoxygenase (ALOX5)*, *P67phox (NCF2)*, *P47phox (NCF1)*, and *P40phox (NCF4)* gene expressions were determined by quantitative real-time polymerase chain reaction (qPCR) following the method described previously [17]. Total RNA was extracted from cells which treated with ox-LDL (80 ng/mL) for 24 h (AS group) and cells which treated with normal saline for 24 h (control group) (extraction kit: Mei5bio, MF036-01). The extracted total RNA was reversely transcribed into cDNA using the PrimeScript RT Master Mix (RR036A, Takara). Amplification was performed using SYBR Green Premix (RR420A, Takara). *β*-Actin was used as the internal reference for mRNA qPCR. Independent-sample *t*-test was used to validate significant differences. *P* < 0.05 was considered statistically significant. The primer sequences of the studied genes are as follows: (forward primer) 5′-GTGCTATGTTGCTCTAGACTTCG-3′ and (reverse primer) 5′-ATGCCACAGGATTCCATACC-3′ for *β-actin*; (forward primer) 5′-ACTACATCTACCTCAGCCTCATT-3′ and (reverse primer) 5′-GATGTGAATTTGGTCATCTCGG-3′ for *ALOX5*; (forward primer) 5′-GAAGATACCTCTCCAGAATCCG-3′ and (reverse primer) 5′-TTCTTAGACACCATGTTCCGAA-3′ for *NCF2*; (forward primer) 5′-ATTCACCGAGATCTACGAGTTC-3′ and (reverse primer) 5′-TGAAGTATTCAGTGAGAGTGCC-3′ for *NCF1*; (forward primer) 5′-ATTCACCGAGATCTACGAGTTC-3′ and (reverse primer) 5′-TGAAGTATTCAGTGAGAGTGCC-3′ for *NCF4*.

Eight-week-old male C57BL/6 mice were fed with a normal diet (control group), and eight-week-old male APOE-/- mice were fed with a high-fat diet (AS group) for 3 months. Aortic valve histological changes were assessed between two groups. The aortic valve specimens were embedded into paraffin, then sectioned into slides, and processed for hematoxylin and eosin (HE) staining and immunohistochemical (IHC) staining. Homogenized arterial tissues were separated onto 10% SDS-PAGE gels, transferred to polyvinylidene difluoride membranes and then probed using the *ALOX5* antibodies (ab169755, Abcam) and *NCF2* antibodies (Cat No. 15551-1-AP, Proteintech), which were diluted with 3% BSA. All animal experiments were conducted in a human manner and according to the Institutional Animal Care Instructions guidelines.

### 2.13. Statistical Analysis

Statistical analyses for data of our experiments were performed with the Prism software (GraphPad Software, La Jolla, CA). Independent-sample *t*-test was used to validate significant differences; ∗∗∗ represents *P* < 0.001, ∗∗ represents *P* < 0.01, and ∗ represents *P* < 0.05.

## 3. Results

### 3.1. Identification of DEGs

**Figure 1:**
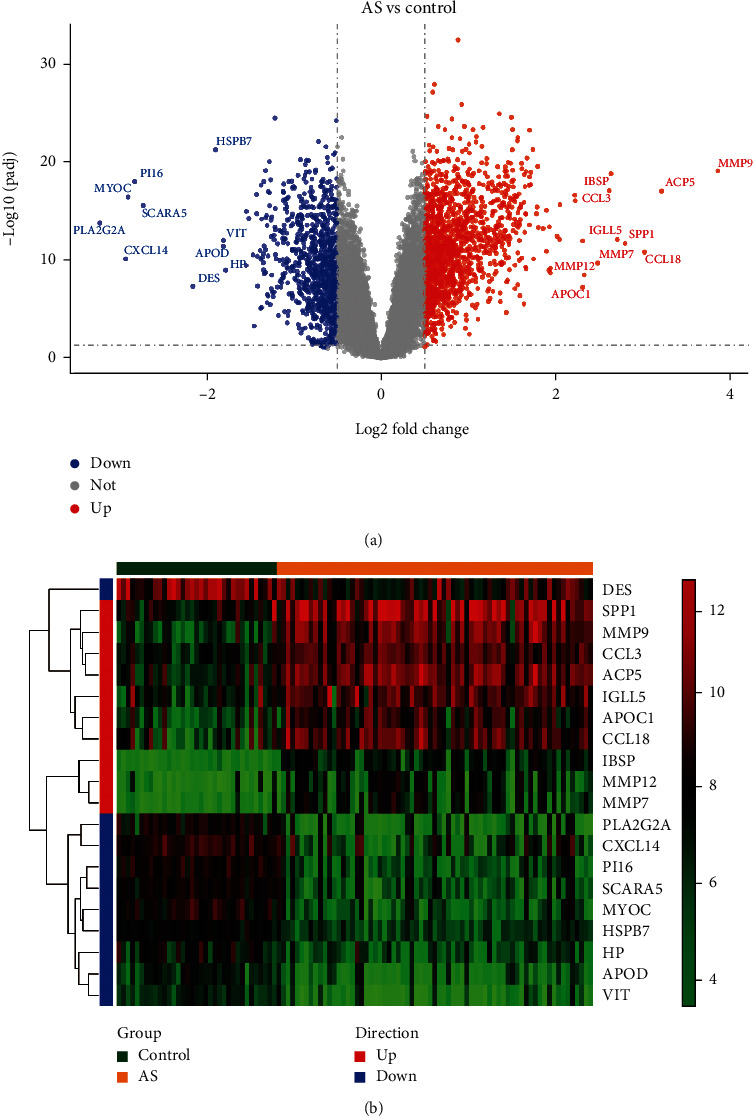
Identification of DEGs. (a) Volcano plot of DEGs. (b) Heatmaps of DEGs.

Differential gene expression analysis was performed using GSE100927. A total of 2417 DEGs (|log2FC| >0.5 and adjusted *P* < 0.05) were obtained, of which 934 DEGs were downregulated and 1483 were upregulated. Volcano and heatmaps of the DEGs are shown in Figures 1(a) and 1(b); the DEGs exhibit significantly different expression patterns between the AS and control samples.

### 3.2. Enrichment Function Analysis of DEOSGs

**Figure 2:**
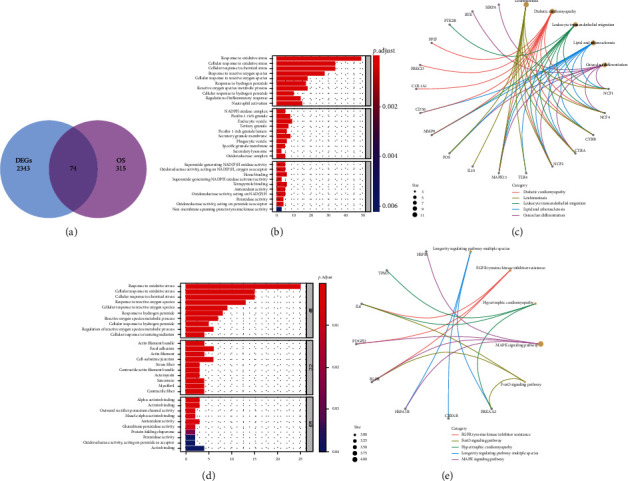
Identification of DEOSGs. (a) Venn diagrams of the DEGs and OS-related genes. (a and b) GO and KEGG analysis of upregulated DEGs. (c and d) GO and KEGG analyses of downregulated DEGs.

A total of 389 OS-related genes were identified by GSEA, and 74 differentially DEOSGs were obtained by intersection with DEGs (Figure 2(a)). The GO analysis results showed that the upregulated DEOSGs were enriched in response to oxidative stress, ficolin-1-rich granule, and antioxidant activity (Figure 2(b)). The downregulated DEOSGs were enriched in response to oxidative stress, focal adhesion, and actin binding (Figure 2(d)). KEGG pathway analysis revealed that the upregulated DEOSGs were mainly enriched in diabetic cardiomyopathy, leishmaniasis, leukocyte transendothelial migration, and atherosclerosis (Figure 2(c)), and the downregulated DEOSGs were mainly enriched in longevity regulating pathway-multiple species, epidermal growth factor receptor (EGFR) tyrosine kinase inhibitor resistance, hypertrophic cardiomyopathy, and MAPK signaling pathway (Figure 2(e)).

### 3.3. Immune Infiltrating Cell Analysis

**Figure 3:**
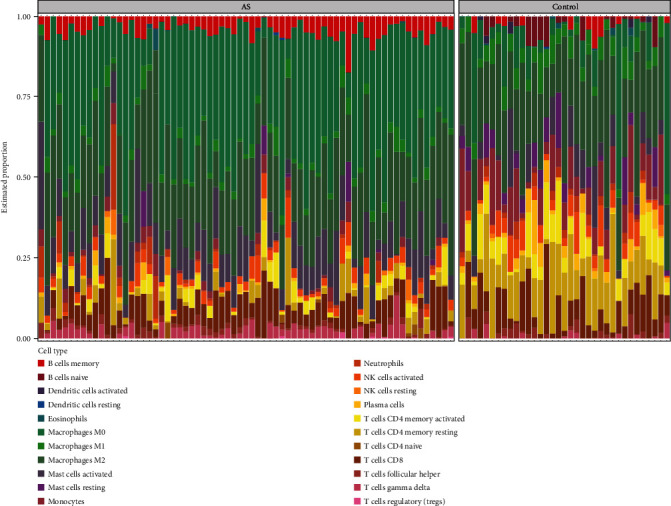
Immune infiltration analysis based on the CIBERSORT algorithm. The enrichment fraction of 22 types of immune infiltrating cells in the AS and normal samples.

**Figure 4:**
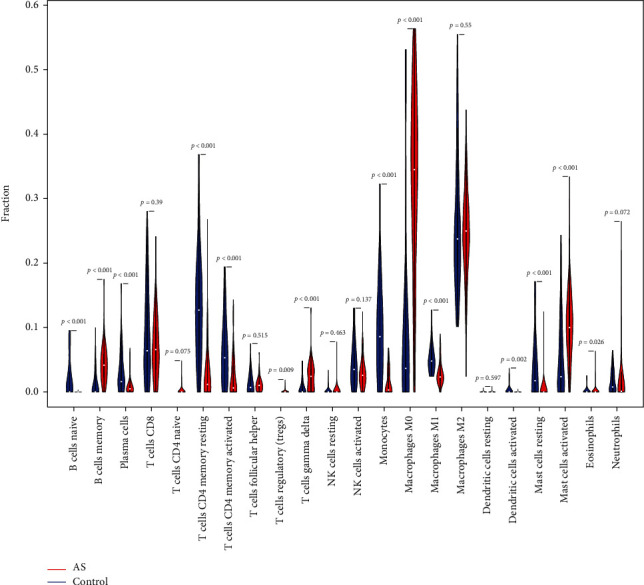
Violin plot of 22 types of immune infiltrating cells in the AS and normal samples.

Figure 3 shows the enrichment fraction of 22 types of immune infiltrating cells in the AS and normal samples. According to Figure 4, 14 types of immune cells were significantly different between the AS and control samples (*P* < 0.05). They included B cells naive, B cells memory, plasma cells, T cells CD4 memory resting, T cells CD4 memory activated, T cells regulatory (Tregs), T cells gamma delta, monocytes, macrophages M0, macrophages M1, dendritic cells activated, mast cells resting, mast cells activated, and eosinophils.

### 3.4. Construction of Coexpression Networks

**Figure 5:**
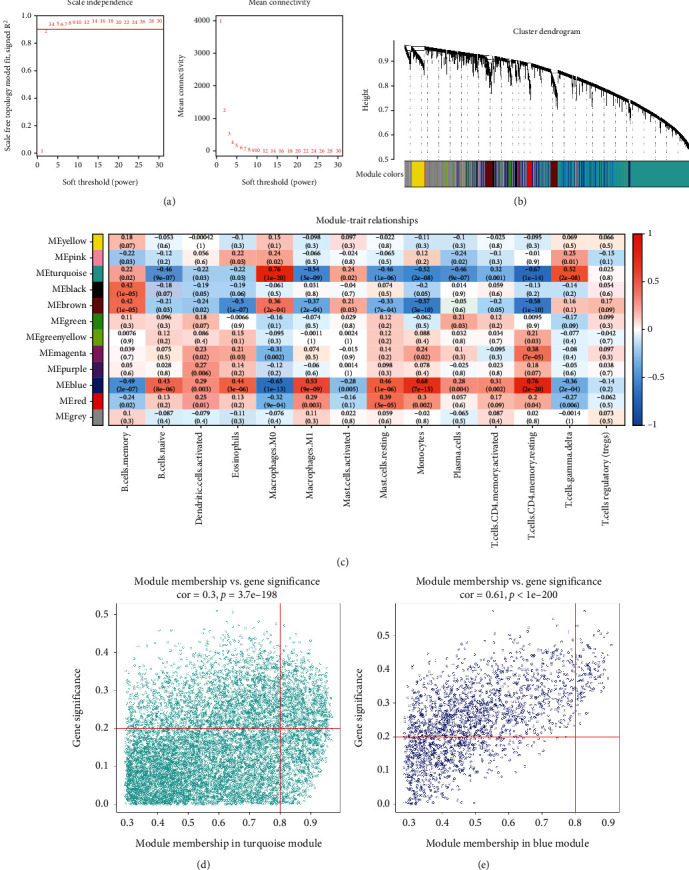
WGCNA coexpression network of immune-related genes. (a) The optimal soft-threshold power. (b) Dendrogram based on a dissimilarity metric for DEGs. (c) Module-trait relationships between WGCNA modules and immune cells. (d–e) Scatter plots of key modules.

The sample cluster tree was shown in Supplement 2(a) and 2(b), and the results indicated that two abnormal samples were removed. Thereafter, the optimal soft-threshold power of 2 was selected based on the scale-free network construction (Figure 5(a)). Finally, through WGCNA analysis, 12 modules were identified in this study (Figure 5(b)). Furthermore, through correlation analysis of the modules and traits (infiltrated immune cells), we found that the turquoise module was highly positively correlated with macrophages M0 (Cor = 0.76, *P* = 1e − 20), and blue module was highly positively correlated with T cells CD4 memory resting (Cor = 0.76, *P* = 2e − 20) (Figure 5(c)). Therefore, the turquoise module and blue module were selected as important modules relevant to immune infiltrating cells for further analysis. In Figures 5(d) and 5(e), the significant correlations between gene significance (GS) and module membership (MM) were presented in the turquoise module and blue modules; 857 and 115 key genes were, respectively, found in the two modules by GS >0.20 and MM >0.80.

### 3.5. Identification of Differentially Expressed Immune-Related Oxidative Stress Genes and Functional Enrichment Analysis

**Figure 6:**
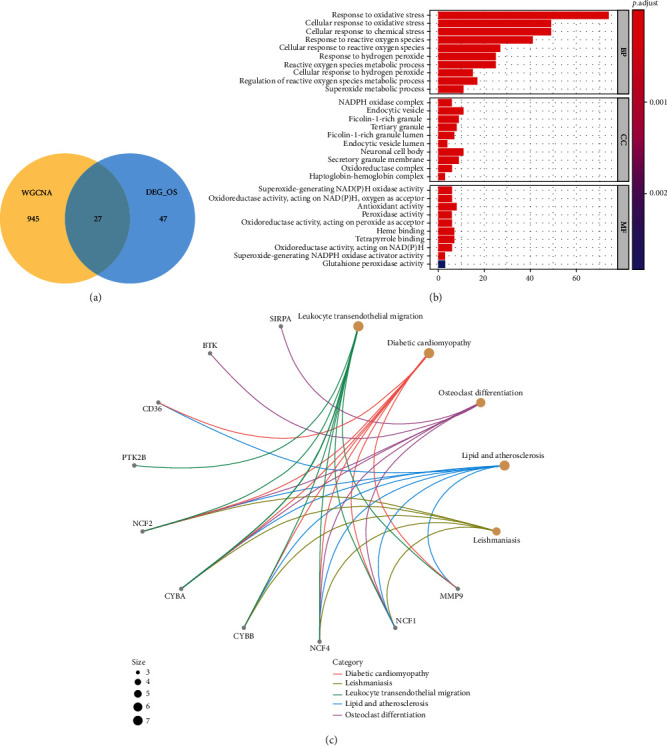
Identification of DEIOSGs. (a) Venn diagrams of the DEOSGs and genes in the blue and turquoise modules. (b) GO analysis of DEIOSGs. (c) KEGG analysis of DEIOSGs.

The intersection between DEOSGs and genes in the blue and turquoise modules was observed. We identified 27 differentially expressed immune-related oxidative stress genes (DEIOSGs) (Figure 6(a)). To explore the function of 27 DEIOSGs in AS, the GO terms are shown in Figure 6(b). In the BP analysis, DEIOSGs mainly participated in response to oxidative stress, cellular response to oxidative stress, and cellular response to chemical stress. In CC analysis, DEIOSGs significantly participated in the neuronal cell body, endocytic vesicle, and secretory granule membrane. MF analysis showed that DEIOSGs significantly participated in antioxidant activity, heme binding, and tetrapyrrole binding. KEGG analysis was performed to explore the pathways of these 27 DEIOSGs. The KEGG terms of DEIOSGs were mainly enriched in leukocyte transendothelial migration, diabetic cardiomyopathy, osteoclast differentiation, atherosclerosis, and leishmaniasis (Figure 6(c)).

### 3.6. Identification of Hub Genes

**Figure 7:**
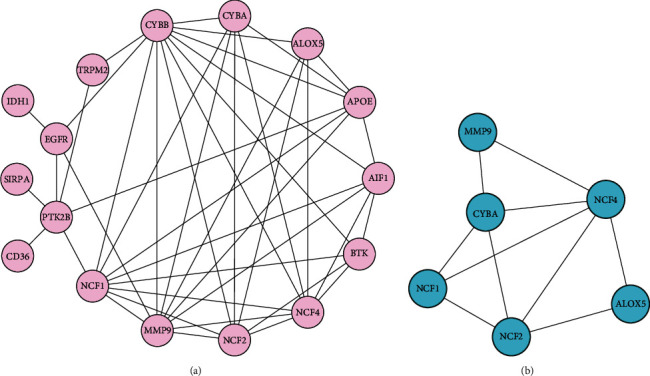
Construction of PPI network and identification of the top module. (a) The PPI network of DEIOSGs. (b) The top1 module in the PPI network.

The PPI network of DEIOSGs was constructed using the STRING and visualized using the Cytoscape (Figure 7(a)). The MCODE of the Cytoscape was used to determine the key genes in the PPI network; six hub genes were obtained, namely *NCF2*, *MMP9*, *ALOX5*, *NCF1*, *NCF4*, and *CYBA* (Figure 7(b)).

### 3.7. The Expression Analysis, ROC Curve Analysis, and Hub Gene Validation for AS Diagnosis

**Figure 8:**
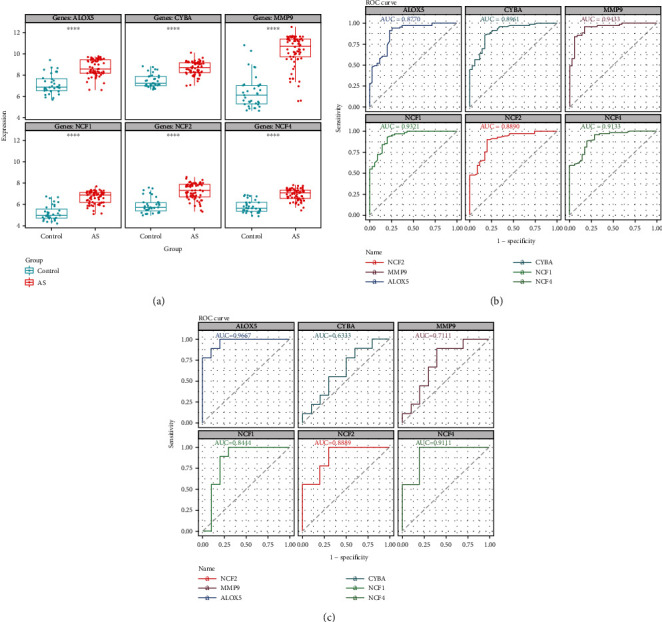
(a) Expression of hub genes in GSE100927. (b) The ROC curve analysis of hub genes in GSE100927. (c) ROC curve analysis of hub genes in the GSE57691, which is the verification set.

We explored the expressions of these genes between AS and control samples in the GSE100927 dataset and found that those genes exhibited higher expression levels in the AS group (Figure 8(a)). In the GSE100927 dataset, to explore the accuracy of the six hub genes as the diagnostic biomarkers for AS, the ROC curves were plotted (Figure 8(b)). Six hub genes with an AUC >0.7 were used as diagnostic markers. Notably, the AUC for *MMP9* was 0.9433, which was the highest among the AUCs of the six hub genes. The other AUC values were 0.8770, 0.8890, 0.8961, 0.9133, and 0.9321 for *ALOX5*, *NCF2*, *CYBA*, *NCF4*, and *NCF1*, respectively. These results showed that the six hub genes had good diagnostic values.

In the external validation set GSE57691, the ROC curves of six hub genes were analyzed. ROC analysis was used to verify the specificity and sensitivity of the six hub genes for AS diagnosis. As shown in Figure 8(c), except for *CYBA*, the AUC areas of all other genes were> 0.7 in the GSE57691 dataset. These results suggest that the five hub genes may serve as diagnostic biomarkers for AS. In addition, the correlation among the five hub genes was analyzed. The results demonstrated that the correlations among the five diagnostic genes were all positive. *NCF2* and *NCF4* had the highest correlation coefficient of 0.95. The results of functional similarity showed that three diagnostic genes, including *NCF2*, *NCF4*, and *NCF1* (similarity score> 0.5), had higher functional similarity (Supplement 3(a) and 3 (b)).

### 3.8. Correlation Analysis between Diagnostic Genes and Immune Cells

**Figure 9:**
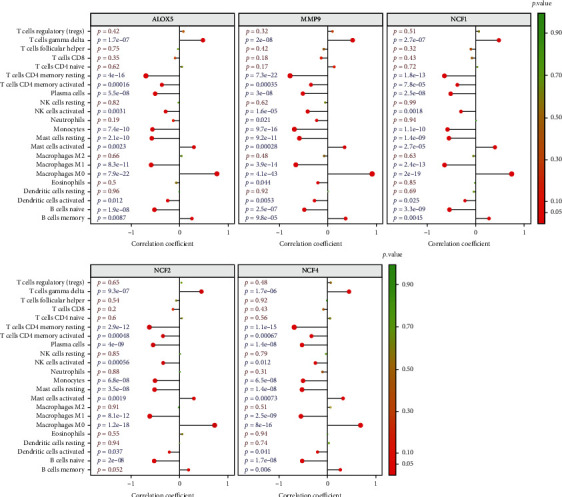
Correlation between *ALOX5*, *NCF2*, *NCF4*, *NCF1*, and *MMP9* with immune infiltrating cells.

To further understand the role of these genes in immune infiltration, we used Spearman correlation analysis to determine whether these diagnostic genes were related to immune cell infiltration. Correlation analysis showed that five diagnostic genes including *ALOX5*, *NCF2*, *MMP9*, *NCF4*, and *NCF1* had significantly positive relationship with the infiltration of T cells gamma delta, mast cells activated, and macrophages M0. Five diagnostic genes had significantly negative relationship with the infiltration of T cells CD4 memory resting, T cells CD4 memory activated, plasma cells, NK cells activated, monocytes, mast cells resting, macrophages M1, dendritic cells activated, and B cells naïve (Figure 9).

### 3.9. GSEA Analysis and Prediction of Key miRNAs, TF, and Drug-Gene Networks

**Figure 10:**
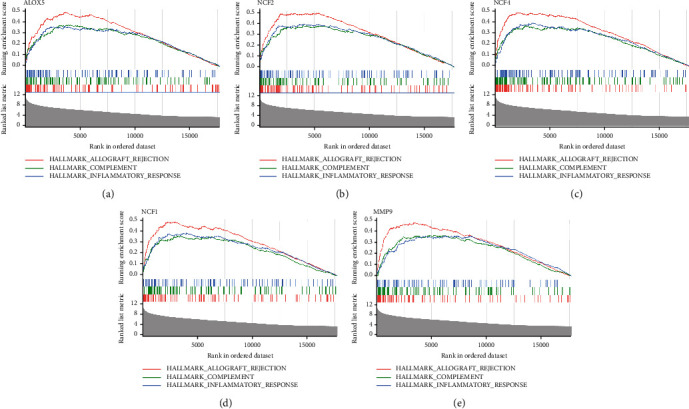
The function of *ALOX5*, *NCF2*, *NCF4*, *NCF1*, and *MMP9* using GSEA analysis (a–e).

The functions of our diagnostic genes were explored using GSEA. Genes in the high-expression cohorts of *ALOX5*, *NCF2*, *MMP9*, *NCF4*, and *NCF1* were all highly enriched in allograft rejection and inflammatory response (Figures 10(a)–10(e)). After considering the results of GSEA, we concluded that these five genes might be highly correlated with inflammation.

**Figure 11:**
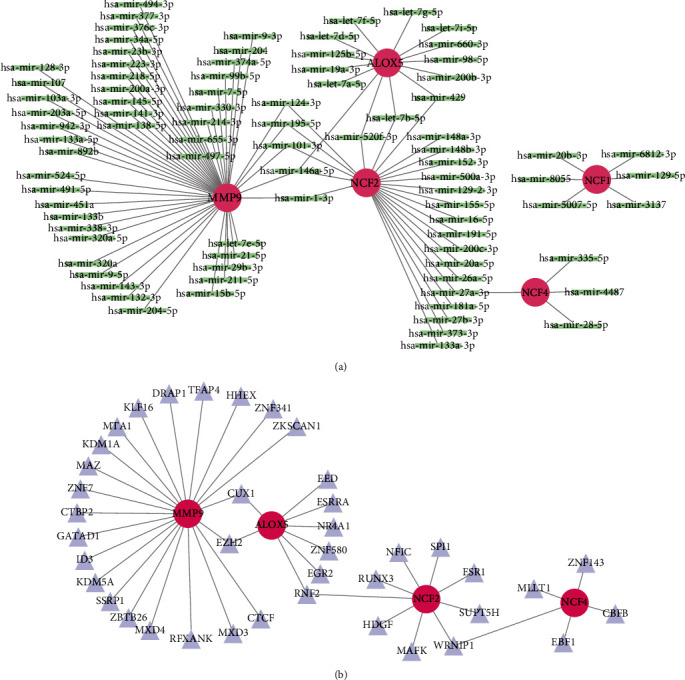
The miRNA network and TF network that regulate the hub genes. (a) The miRNA network of *ALOX5*, *NCF1*, *NCF2*, *NCF4*, and *MMP9*. (b) The TF network of *MMP9*, *ALOX5*, *NCF2*, and *NCF4*.

Four online databases were accessed to predict potential miRNA targeting diagnostic genes, and the miRNA-diagnostic gene regulatory network is shown in Figure 11(a).

The interaction network consisted of four diagnostic genes and 40 TFs (Figure 11(b)). Twenty-two TFs, including KLF16, DRAP1, and TFAP4, could regulate *MMP9*. Eight TFs, including EED, ZNF580, and EGR2, could regulate *ALOX5*. Nine TFs, including ESR1, MAFK, and HDGF, could regulate *NCF2*. Five TFs, including EBF1, CBFB, and MLLT1, could regulate *NCF4*. Twenty-one potential drugs for treating patients with AS were identified when the drug-gene interactions were explored using CTD. Additionally, drug-gene networks were constructed using the Cytoscape (Supplement 4).

### 3.10. Single-Cell Analysis

**Figure 12:**
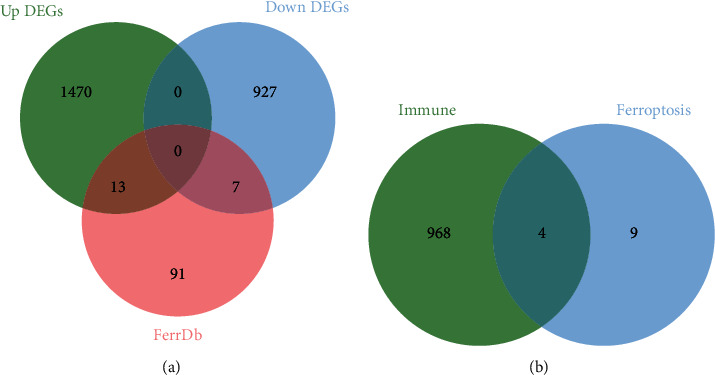
Identification of upregulated ferroptosis-related DEGs. (a) Venn diagrams of the upregulated DEGs, downregulated DEGs, and ferroptosis gene set. (b) Venn diagrams of the upregulated ferroptosis-related DEGs and immune-related genes (the turquoise module genes and the blue module genes.

We respectively took the intersection of upregulated DEGs and downregulated DEGs with ferroptosis gene set, pyroptosis gene set, and necroptosis gene set. Thirteen upregulated ferroptosis-related DEGs, ten upregulated necroptosis-related DEGs, and eight upregulated pyroptosis-related DEGs were identified (Figure 12(a), Supplement Figures 5(a) and 5(b)). Among them, *ALOX5* and *NCF2* genes were upregulated ferroptosis-related DEGs. Additionally, the intersection between upregulated ferroptosis-related DEGs and genes in the blue and turquoise modules was assessed. Finally, we identified four upregulated immune-related ferroptosis genes: *ALOX5*, *NCF2*, AURKA, and SLC2A6 (Figure 12(b)).

**Figure 13:**
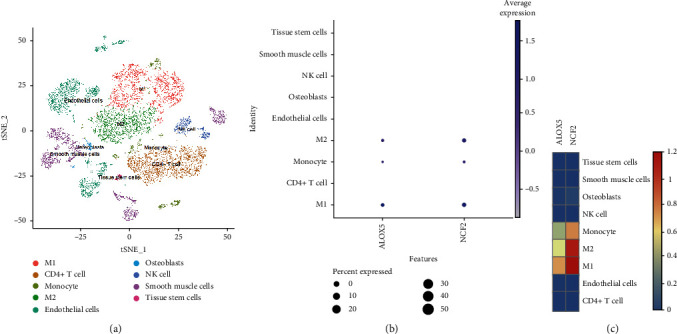
Single-cell analysis identified the expression of *ALOX5* and *NCF2*. (a) Umap of nine cell types in GSE159677. (b and c) *ALOX5* and *NCF2* had high expression levels in monocyte and macrophages M1 and macrophages M2.

Combined with single-cell analysis, we identified the distribution of *ALOX5* and *NCF2* genes in the nine integrated cell populations. The results further confirmed that *ALOX5* and *NCF2* were significantly highly expressed in macrophages M1, macrophage M2, and monocytes (Figure 13(a)–13(c)).

### 3.11. In Vitro and In Vivo Analyses

**Figure 14:**
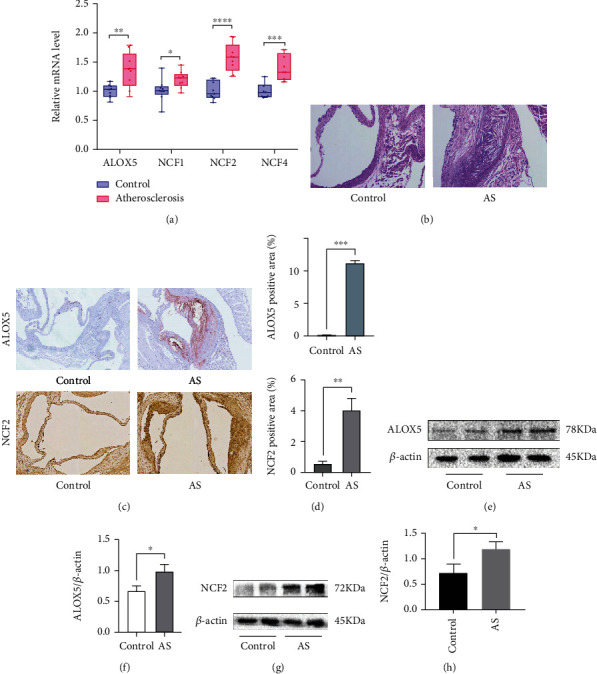
In vitro and in vivo experiments verified the expression of hub genes. (a) Relative mRNA level of *ALOX5*, *NCF1*, *NCF2*, and *NCF4* in RAW264.7. (b) HE stains showed control group in C57BL/6 mice and AS group in APOE-/- mice. (c and d) Immunohistochemistry staining of *ALOX5* and *NCF2* in the aortic valve. Analysis of *ALOX5*-positive area and *NCF2*-positive area in immunohistochemistry stains. (e, g) Increased *ALOX5* and *NCF2* expression in AS tissues and control tissues were detected by western blotting. (f, h) Analysis of *ALOX5* and *NCF2* protein relative expression in AS tissues compared to the control tissues, ∗∗∗*P* < 0.001, ∗∗*P* < 0.01, and ∗*P* < 0.05.

Compared with the control group (normal saline treated group), the AS group (OX-LDL-treated group) had increased relative mRNA expression of *ALOX5* (*P* = 0.0071), *NCF1* (*P* = 0.0336), *NCF2* (*P* < 0.0001), and *NCF4* (*P* = 0.0002) (Figure 14(a)). HE stains revealed larger aortic valve plaque formation areas in APOE-/- mice fed with high fat than C57BL/6 mice fed with normal diet. And there was necrotic core within the plaque (Figure 14(b)). Furthermore, we used IHC to preliminarily detect the expression of *ALOX5* and *NCF2* in aortic valve plaque tissues of mice (Figure 14(c)). The positive areas of *ALOX5* and *NCF2* were higher in the AS group than the control group (Figure 14(d)). The western blot results further confirmed that *ALOX5* and *NCF2* were more highly expressed in the AS group than in the control group (Figures 14(e)–14(h)).

## 4. Discussion

Inflammatory responses and the modification of lipoproteins that cause lipid accumulation in AS are associated with imbalance of oxidative stress and immune processes [18]. However, few studies have focused on the aberrantly expressed gene biomarkers associated with immune infiltration and oxidative stress between AS and normal tissues. Herein, we identified 27 DEIOSGs. The enrichment analysis revealed that DEIOSGs were primarily engaged in cellular response to oxidative stress, NADPH oxidase activity, and atherosclerosis. *MMP9*, *ALOX5*, *NCF2*, *NCF1*, and *NCF4* were identified as diagnostic biomarkers of AS using two GEO datasets. Additionally, we observed a dramatic difference in immune cell content between AS and normal samples. *MMP9*, *ALOX5*, *NCF2*, *NCF1*, and *NCF4* were mainly associated with macrophages. Previous studies have demonstrated that macrophage-derived *MMP9* promotes the infiltration of monocyte/macrophages into the lesions thereby enhancing atherosclerosis [19] and *NCF1* expression leads to priming of human macrophage oxidative burst [20]. However, fewer studies have focused on how these genes regulate immune cells in atherosclerosis. Oxidative stress promotes the upregulation and accumulation of ox-LDL in macrophages and the formation of foam cells [21]. We confirmed that four pivotal OS-related genes (*ALOX5*, *NCF2*, *NCF1*, and *NCF4*) were significantly high expression in ox-LDL-treated macrophages.

Shen et al. [22] exhibited that the atheromatous plaques in the ImmuneScoreH cluster (most plaques from the carotid arteries) had higher proportions of M0 macrophages than ImmuneScoreL cluster (most plaques from the infrapopliteal and femoral arteries). It indicates that there existed distinct heterogeneity of immune infiltration in different atherosclerotic lesions. So, we explored immune infiltration of 69 atheromatous plaques from different arterial beds in GSE100927 by CIBERSORT algorithm. Our results indicated that atheromatous plaques in GSE100927 (most plaques from the carotid arteries) had higher proportions of M0 macrophages than control samples, which was consistent with previous study results [22]. In addition, we found that the atheromatous plaques had less proportions of M1 macrophages than control samples in GSE100927. This finding suggested that macrophage death might be at the stage of macrophage polarization in atherosclerosis. Previous study also showed that macrophage death is a major contributor to necrotic core formation and plaque destabilization [23].

Recently, emerging evidence has implicated the critical role of programmed cell death pathways in macrophage foam cells loss, including necroptosis, pyroptosis, and ferroptosis [23]. As a result, we compared upregulated DEGs that associated with necroptosis, pyroptosis, and ferroptosis. There were thirteen upregulated ferroptosis-related DEGs. Compared with the necroptosis and pyroptosis, ferroptosis had the greatest number of upregulated DEGs, indicating that ferroptosis may has a major role in cell death of atherosclerotic plaque. The correlation analysis showed that ferroptosis-related *ALOX5* and *NCF2* were positively associated with macrophages M0 and negatively associated with macrophages M1 in AS. The further single-cell analysis also confirmed that *ALOX5* and *NCF2* were mainly expressed in macrophage M1. These findings suggest that *ALOX5* and *NCF2* may regulate macrophage ferroptosis by mediating oxidative stress in the stage of polarization. *NCF2 (p67phox)* had been identified as potential diagnostic biomarkers in patients with obstructive coronary artery [24] and psoriasis complicated with atherosclerosis [25]. Our study not only confirmed that *NCF2* was significantly overexpressed in atherosclerotic plaques, but also confirmed for the first time that *NCF2* was mainly correlated with macrophage of plaques. In previous studies, there was no direct evidence indicated that *NCF2* was related to ferroptosis, but ferroptosis could be triggered by reactive oxygen species (ROS) under the activation of nicotinamide adenine dinucleotide phosphate (NADPH) oxidase (NOXs) [26]. *NCF2* is a core subunit of NOXs, which is needed to active the complex [24]. The NOXs inhibitor diphenyleneiodonium and the NOX1/4-specific inhibitor GKT137831 prevent erastin-induced ferroptosis in Calu-1 and HT1080 cells [27], suggesting that members of the NOX family promote ferroptosis of cancer cells [28–30]. Besides, previous study confirmed that *ALOX5* was a key enzyme for evoking ferroptosis in cancer diseases [31] or neuronal injury [32]. *5-lipoxygenase (ALOX5)* can catalyze arachidonic acid (AA) to generate proinflammatory cytokine leukotrienes (LTs) and can also produce lipid peroxides and mediate lipid peroxidation; phospholipids, the main component of cell membrane, are prone to lipid peroxidation, resulting in membrane rupture and ferroptosis [33]. But there was no direct evidence indicated that *ALOX5* mediated ferroptosis in atherosclerotic plaques. Mehrabian et al. [34] only exhibited that *ALOX5* deficiency (*ALOX5* (-/-)) mice protects against atherosclerosis. Consistent with that, this study found that atherosclerotic plaques had higher expression of ALOX5. And we further confirmed that macrophage-derived foam cells had higher expression of ALOX5. In general, the innovation of this study is the exploration of pivotal OS-related genes and ferroptosis-related genes associated with macrophages in atherosclerosis using multibioinformatic analyses and experiments. Previous outstanding single-cell proteomic and transcriptomic studies of correlation between OS and macrophages ferroptosis were mainly studied in cancer diseases [35, 36]. However, the study of plaques has only analyzed OS or ferroptosis [37] separately. To the best of our knowledge, this was the first bioinformatics report describing the coexistence of immune cell ferroptosis and oxidative stress in AS. This study also has several limitations. Firstly, more experiments are required to provide the direct evidence of macrophage ferroptosis. Secondly, immune infiltration analysis was performed based on transcriptomic data. Hence, we could not determine if ferroptosis caused macrophages polarization, or whether polarized macrophages happen ferroptosis. Further studies are warranted to clarify the underlying mechanisms.

## 5. Conclusion

Our results provided novel targets for predicting atherosclerotic plaque progression and confirmed that *ALOX5* and *NCF2* have good diagnostic value for atherosclerosis. We predicted that ferroptosis of macrophages may become the potential target in atherosclerosis. However, additional factors also need to be combined to develop effective strategies for preventing cardiovascular events.

## Data Availability

The analysis data used to support the findings of this study are available from the corresponding author upon request.

## Supplementary Materials

Supplementary Materials Supplement Figure 1: technology road mapping of this article. Supplement Figure 2: Sample clustering of WGCNA to detect outliers (a and b). Supplement Figure 3: (a) The correlations of MMP9, ALOX5, NCF2, NCF1, and NCF4. (b) The functional similarity of MMP9, ALOX5, NCF2, NCF1, and NCF4. Supplement Figure 4: PPI network between small-molecule drug with MMP9, ALOX5, NCF2, NCF1, and NCF4. Supplement Figure 5: identification of pyroptosis-related DEGs and necroptosis-related DEGs. (a) The intersection of upregulated DEGs and downregulated DEGs with pyroptosis-related genes. (b) The intersection of upregulated DEGs and downregulated DEGs with necroptosis-related genes.
