# Supplementary material for: Novel Diagnostic Biomarkers Related to Oxidative Stress and Macrophage Ferroptosis in Atherosclerosis
Source: Oxid Med Cell Longev. 2022 Aug 5;2022:8917947. doi: 10.1155/2022/8917947 (PMC9410850; doi:10.1155/2022/8917947)
Supplement: Supplementary Materials — Supplement Figure 1: technology road mapping of this article. Supplement Figure 2: Sample clustering of WGCNA to detect outliers (a and b). Supplement Figure 3: (a) The correlations of MMP9, ALOX5, NCF2, NCF1, and NCF4. (b) The functional similarity of MMP9, ALOX5, NCF2, NCF1, and NCF4. Supplement Figure 4: PPI network between small-molecule drug with MMP9, ALOX5, NCF2, NCF1, and NCF4. Supplement Figure 5: identification of pyroptosis-related DEGs and necroptosis-related DEGs. (a) The intersection of upregulated DEGs and downregulated DEGs with pyroptosis-related genes. (b) The intersection of upregulated DEGs and downregulated DEGs with necroptosis-related genes. [file 8917947.f1.pdf]

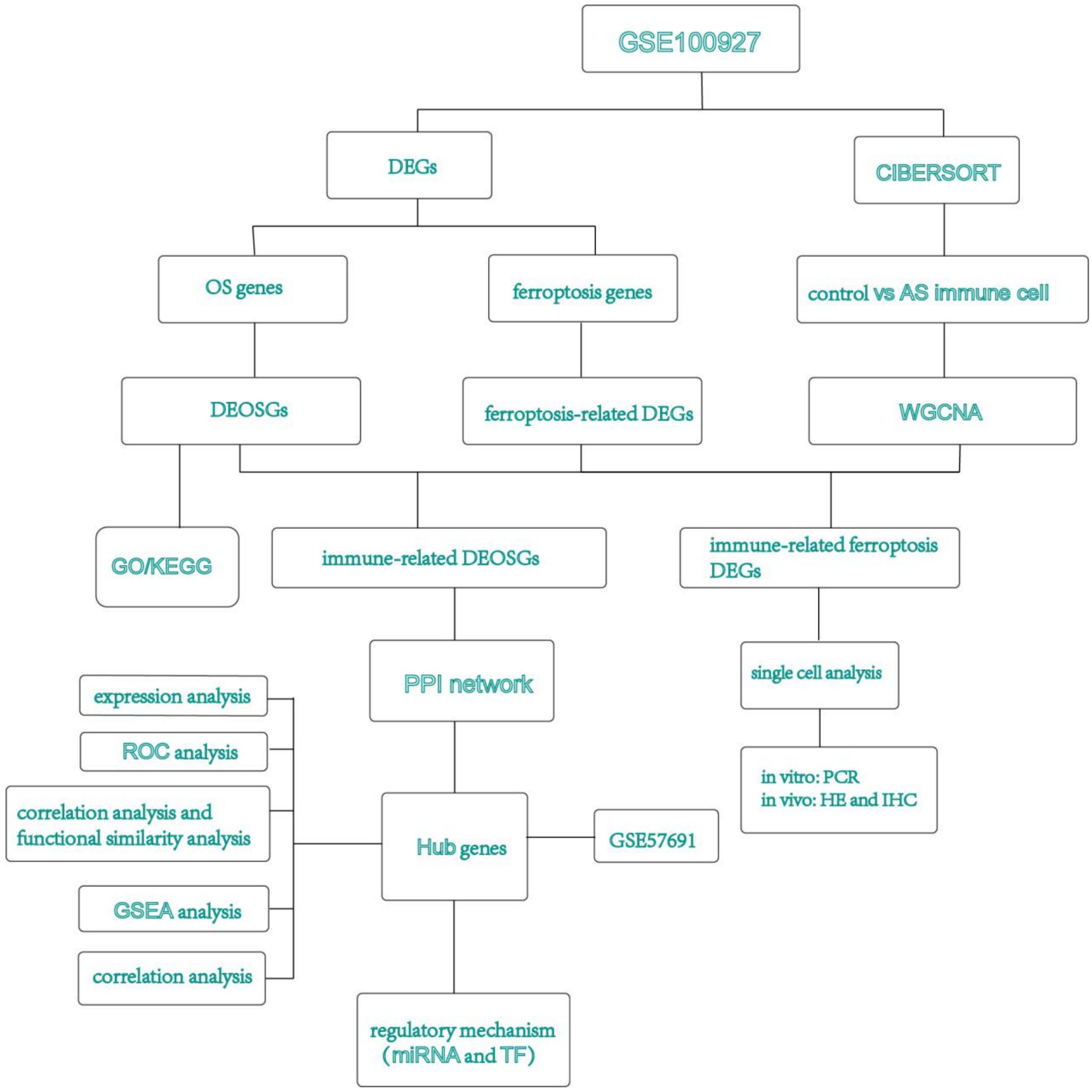


Supplement figure 1. Technology road mapping of this article.

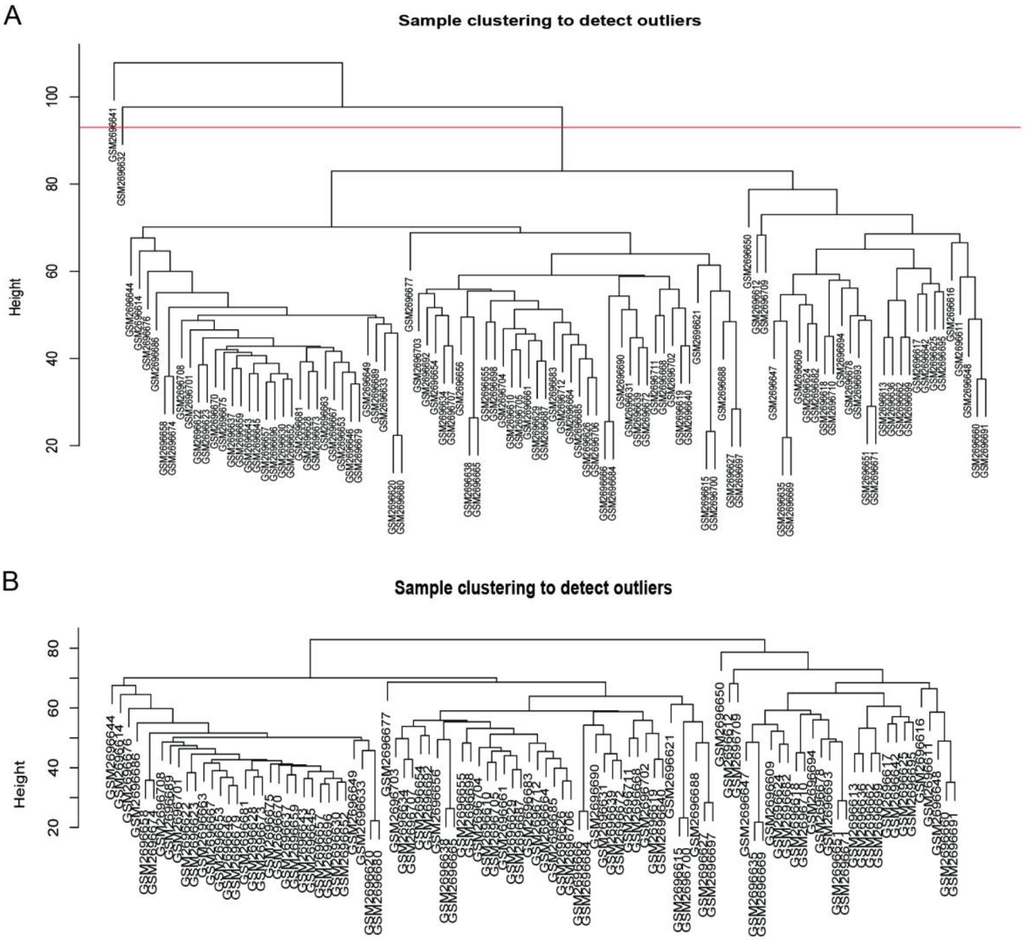


Supplement figure 2. Sample clustering of WGCNA to detect outliers (A-B).

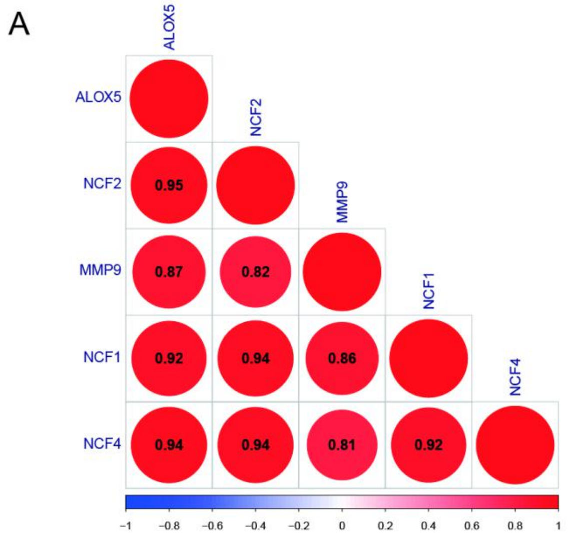


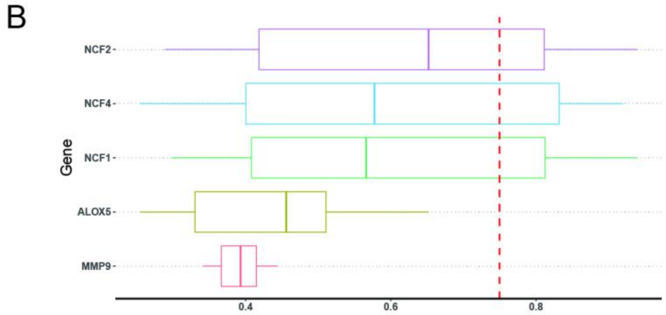


Supplement figure 3. (A) The correlations of MMP9, ALOX5, NCF2, NCF1 and NCF4. (B) The functional similarity of MMP9, ALOX5, NCF2, NCF1 and NCF4.

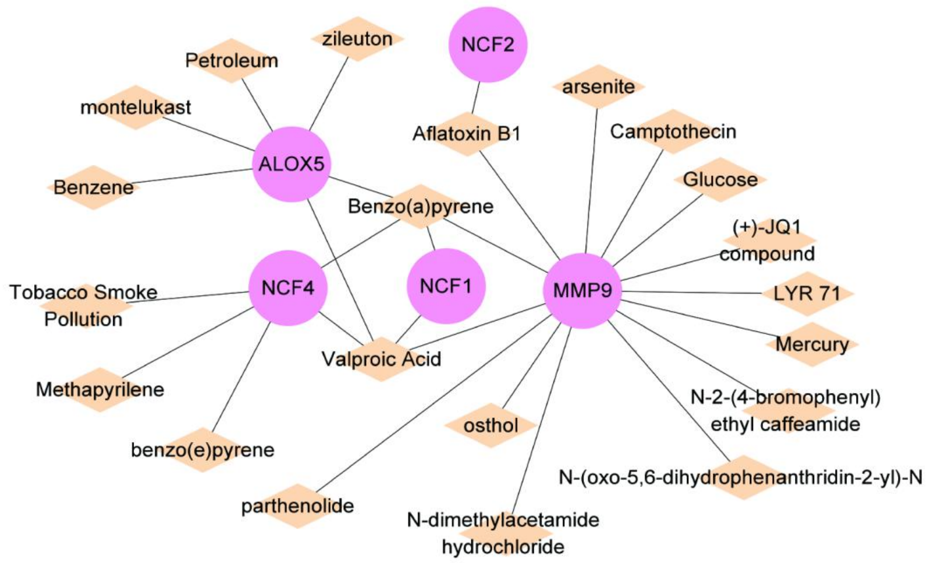


Supplement figure 4. PPI network between small-molecule drug with MMP9, ALOX5,

NCF2, NCF1 and NCF4.

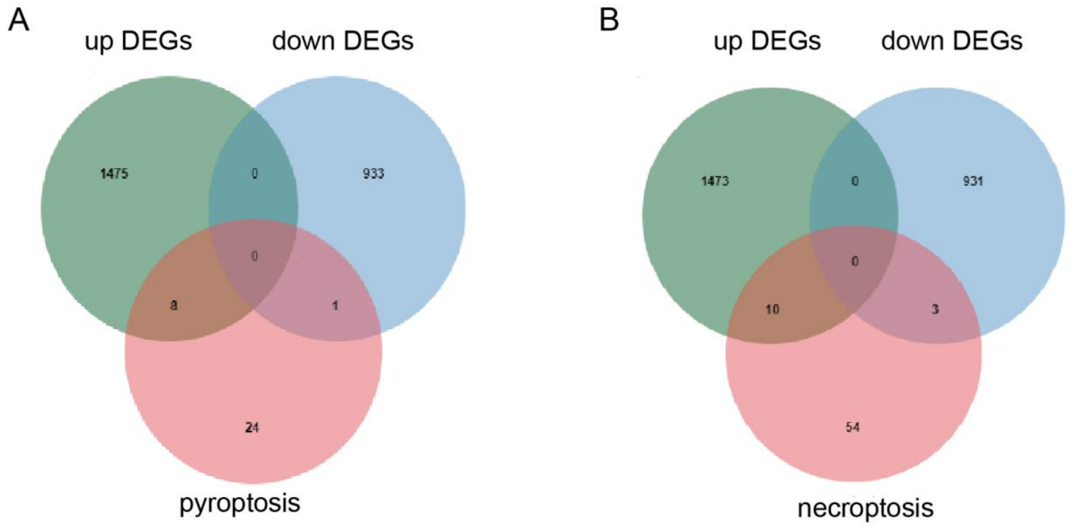


Supplement figure 5. Identification of pyroptosis-related DEGs and necroptosis-related DEGs. (A) The intersection of upregulated DEGs, downregulated DEGs with pyroptosis-related genes. (B) The intersection of upregulated DEGs, downregulated DEGs with necroptosis-related genes.
